# CRISP-R/Cas9 Mediated Deletion of Copper Transport Genes CTR1 and DMT1 in NSCLC Cell Line H1299. Biological and Pharmacological Consequences

**DOI:** 10.3390/cells8040322

**Published:** 2019-04-06

**Authors:** Ekaterina Y. Ilyechova, Elisa Bonaldi, Iurii A. Orlov, Ekaterina A. Skomorokhova, Ludmila V. Puchkova, Massimo Broggini

**Affiliations:** 1 Laboratory of Trace elements metabolism, ITMO University, Kronverksky av. 49, 197101 St.-Petersburg, Russia; ikaterina2705@yandex.ru (E.Y.I.); orlov239@gmail.com (I.A.O.); katjaskom@yandex.ru (E.A.S.); massimo.broggini@marionegri.it (M.B.); 2 Department of Molecular Genetics, Research Institute of Experimental Medicine, Acad. Pavlov str. 12, 197376 St.-Petersburg, Russia; 3 Department of Biophysics, Peter the Great St. Petersburg Polytechnic University, Politekhnicheskaya str. 29, 195251 St.-Petersburg, Russia; 4 Laboratory of molecular pharmacology, Istituto di Ricerche Farmacologiche “Mario Negri”, IRCCS, Via La Masa 19, 20156 Milan, Italy; eli.bon89@gmail.com

**Keywords:** copper importers CTR1 and DMT1, CRISPR-Cas9, cisplatin, silver, signaling, cuproenzymes, copper homeodynamics

## Abstract

Copper, the highly toxic micronutrient, plays two essential roles: it is a catalytic and structural cofactor for Cu-dependent enzymes, and it acts as a secondary messenger. In the cells, copper is imported by CTR1 (high-affinity copper transporter 1), a transmembrane high-affinity copper importer, and DMT1 (divalent metal transporter). In cytosol, enzyme-specific chaperones receive copper from CTR1 C-terminus and deliver it to their apoenzymes. DMT1 cannot be a donor of catalytic copper because it does not have a cytosol domain which is required for copper transfer to the Cu-chaperons that assist the formation of cuproenzymes. Here, we assume that DMT1 can mediate copper way required for a regulatory copper pool. To verify this hypothesis, we used CRISPR/Cas9 to generate H1299 cell line with *CTR1* or *DMT1* single knockout (KO) and *CTR1*/*DMT1* double knockout (DKO). To confirm KOs of the genes qRT-PCR were used. Two independent clones for each gene were selected for further studies. In CTR1 KO cells, expression of the *DMT1* gene was significantly increased and vice versa. In subcellular compartments of the derived cells, copper concentration dropped, however, in nuclei basal level of copper did not change dramatically. CTR1 KO cells, but not DMT1 KO, demonstrated reduced sensitivity to cisplatin and silver ions, the agents that enter the cell through CTR1. Using single CTR1 and DMT1 KO, we were able to show that both, CTR1 and DMT1, provided the formation of vital intracellular cuproenzymes (SOD1, COX), but not secretory ceruloplasmin. The loss of CTR1 resulted in a decrease in the level of COMMD1, XIAP, and NF-κB. Differently, the DMT1 deficiency induced increase of the COMMD1, HIF1α, and XIAP levels. The possibility of using CTR1 KO and DMT1 KO cells to study homeodynamics of catalytic and signaling copper selectively is discussed.

## 1. Introduction

In mammals, copper has two essential physiological functions. First, it is a catalytic and structural cofactor of enzymes necessary for respiration, antioxidant protection, post-translational modification of neuropeptides, for the synthesis of neurotransmitters, the formation of collagen and elastin and iron transport [1,2]. Second, copper inside and outside the cell is required for the activity of some regulatory proteins (HIF1α, XIAP, COMMD1, NF-κB, p53) which are involved in signaling pathways [3,4,5,6,7]. The indispensable biological functions of copper are inseparable from its high toxicity, which is compensated by an intricate system of carriers that coordinate copper through specific sites, and safely transfer it from the extracellular space to the cell places of cuproenzyme formation (mitochondria, Golgi apparatus, cytosol) [8].

It is still unclear how copper is recruited into signaling pathways, or how it is secreted from the cell to participate in neovascularization [9]. As a non-sophisticated version of this issue, it can be assumed that these copper streams enter the cell through different gates. The main universal importer of copper in mammalian cells is CTR1 [10,11]. It is a transmembrane homotrimer, each subunit of which contains three α-helices, forming a transmembrane domain of nine α-helixes. The N-terminal extracellular domain contains three Cu(II)/Cu(I) sites. In a homotrimer, N-termini form a high-affinity copper trap from the environment, where its concentration is low. The short C-terminal domain contains a copper-binding HCH-motif. All cytosolic copper carriers required for cuproenzymes, pick up the delivered copper, due to a protein–protein interaction with C-domain CTR1 [12]. One might think that catalytic copper enters through the CTR1. 

The cells contain another importer, also capable of carrying copper. It is the divalent metal transporter 1 (DMT1), also known as divalent cation transporter 1 (DCT1) and natural resistance-associated macrophage protein 2 (NRAMP 2), the member 2 of solute carrier family [13]. It consists of a single subunit. Putative topological DMT1 model predicts that DMT1 is a type III integral membrane protein (both N- and C-terminus are oriented to the cytoplasm), with a transmembrane domain from 12 α- helixes and two sites for core N-glycosylation in the extracellular loop between seven and eight α- helixes [14]. It is well established and it functions as a proton-coupled pump by using the cell membrane potential for active transport. DMT1 imports iron as Fe(II), and also Mn(II), Zn(II), Cu(II), Ni(II), Co(II), Pb(II) and Cd(II) ions. It is ubiquitously expressed, most notably in the apical membrane of the enterocytes in duodenum [13]. The cation binding peptide is formed by DMT1-α-helix1 as an α-helix-extended segment–α-helix configuration, in which the negatively charged motif Asp-Pro-Gly-Asn responsible for cation binding is located at the central flexible region [15]. 

DMT1 transports copper in both Cu (II)/Cu (I) oxidation states [16], and even when the *CTR1* gene is switched off, it compensates for its deficiency [17]. C-terminal domain of DMT1 has no homology with C-terminus of CTR1, so, it is unlikely that it is capable of transferring copper to cuproenzyme-associated Cu-transporters. It is yet unknown which proteins, or low molecular weight substances, take copper from DMT1. It is possible that DMT1 represents the pathway for regulatory copper. To check this hypothesis, we obtained single knockout CTR1 and DMT1 cells as well as a double knockout of both genes. The effect of *CTR1* and *DMT1* deficiency on intracellular copper and silver distribution was studied. Engineered cells were tested for resistance to cisplatin and silver ions, and to some additional drugs acting with different mechanisms of action. The expression profile of gene-coding copper-requiring proteins was also determined.

## 2. Materials and Methods

### 2.1. Cell Lines

The human non-small cell lung cancer cell line H1299 (WT) was used for these experiments. These cells do not express the tumor suppressor p53 protein. They were grown in RPMI medium supplemented with 10% fetal bovine serum (FBS). Cells were transfected with CRISPR-Cas9 KO plasmids with three targets specific guide RNAs (gRNA) of 20 nt (for both CTR1 and DMT1 genes). The plasmids were co-transfected with homology-directed repair (HDR) plasmids specific for each gene (Santa Cruz Biotechnology, Santa Cruz, CA, USA), which allow the insertion of puromycin resistance gene and red fluorescent protein (RFP) gene during the repair process. Puromycin and RFP genes can then be removed using Cre recombinase, thanks to the presence of loxP sites in HDR plasmids.

Cells were seeded at high density 24 h before transfection in six-well plates. Fugene (Promega, Madison, WI, USA) was used as transfection reagent. Twenty-four hours after transfection, cells were detached and seeded in 10 mL Petri dishes at a density of 500 cells/plate. After the following 48 h, puromycin (2 µg/mL) was added to allow selection of positive clones. A double selection of clones growing in puromycin-containing medium and expressing RFP was performed. Positive clones were isolated and transferred to six-well plates. 

Expression of CTR1 or DMT1 mRNA by qRT-PCR was used to confirm the KO of the genes. Two independent clones for each gene were selected for further studies.

For the generation of double KO cells (for both CTR1 and DMT1 genes) one clone deleted in CTR1 was treated with Cre recombinase to remove puromycin and RFP genes and subjected to the second round of transfection using a mix of DMT1 KO plasmid and DMT1 HDR plasmid using the same procedure as described for single KO generation. 

### 2.2. Cell Growth and Cytotoxicity

The growth in vitro of the different clones was determined using the RealTime GLO system (Promega). All the procedures were performed according to the manufacturing instructions. Luminescence was detected at 24 h interval using the GloMax plate reader (Promega). For each clone, six independent samples were assessed, and the mean doubling time calculated from the linear part of the growth curve for each cell line. The growth inhibitory activity of the various drugs was determined by using the MTS test. Briefly, cells were seeded in 96-well plates and after 24 h treated with increasing concentrations of the drugs for further 72 h. Survival curves were plotted as percentages of untreated controls. At least six replicates for each time point were used and the results represent the average mean and SD of at least three independent experiments.

### 2.3. Reverse Transcription and Real Time Polymerase Chain Reaction (qRT-PCR)

Total RNA was extracted from exponentially growing cells using Maxwell RSC simply RNA cells kit (Promega, Madison, WI, USA) and reverse-transcribed to cDNA using high capacity cDNA reverse transcription kit (Life Technologies, Carlsbad, CA, USA). *DMT1*,* CTR1*, *CTR2*, *XIAP*, *HIF1α*, *NF-κB*, *COMMD1*, *CCS*, and *SOD1* genes expression levels were determined by qRT-PCR performed with Sybr Green PCR master mix (Applied Biosystem, Foster City, CA, USA). Primers were designed according to program “Primer-BLAST” (version 4.1.0, NCBI, USA) and their primary structures are given in Table 1. 

For each gene and each sample, the dissociation curve was evaluated. Samples were then normalized using the expression of the housekeeping gene (actin), and the levels in the KO clones were compared to parental cells. qRT-PCR was performed using the 7900HT sequence detection system (Applied Biosystem, Foster City, CA, USA). Average values from at least three independent experiments are presented.

### 2.4. Mitochondrial DNA (mtDNA) PCR

Samples of the subcellular fractions were treated with 30 µL lysing mixture including 0.5% SDS, 10 µM dithiothreitol, 0.07 µg/µL proteinase K during 2 h at 55 °C. PCR was performed in 30 µL mixture containing 40 pmol. of each primer, 0.2 mM dNTP’s, TaqPol 0.05 U/µL and 3 mM MgCl_2_. Primes (Forward: 5′-aatgaatgtctgcacagc-3′ and Reverse: 5′-gctaggaccaaacctatttg-3′, annealing temperature 55 °C) to D-loop human mtDNA region were used. 

PCR profile: 94 °C for 2 min, 35 cycles from 94 °C for 2 min, 55 °C for 1 min, 72 °C for 1 min, 72 °C 10 min. PCR was optimized using MJ Mini Personal Thermal Cycler (BioRad, Hercules, CA, USA). PCR products were analyzed in a 1.4% agarose gel with ethidium bromide.

### 2.5. Isolation of Subcellular Fractions

Subcellular fractions were isolated by differential centrifugation from cells collected from T75 flask with full growth. Cells were homogenized (1:6 *w*/*v*, respectively) in 250 mM sucrose, prepared in buffer A, containing 100 mM KCl, 5 mM MgCl_2_, 10 mM Tris-HCl (pH 7.4), 5 mM DTT, and 0.5 µL/mL of protease inhibitor cocktail (Sigma, St. Louis, MO, USA), using T10 basic homogenizer for 3 × 20 s at maximum power (IKA, Staufen im Breisgau, Germany). The homogenate was centrifuged at 800× *g* for 10 min. A crude mitochondrial fraction was isolated from the post-nuclear supernatant as sediment after centrifugation at 12,000× *g* for 20 min. An intracellular membrane fraction (ICM), including of the endoplasmic reticulum and Golgi complex was isolated from the post-mitochondrial supernatant as sediment at 23,000× *g*, 60 min. The supernatant of the last centrifugation comprised the cytosolic fraction. Centrifugation was performed at +4 °C in Allegra X-30 (Beckman, Brea, CA, USA). The pellet obtained by first centrifuging of cellular homogenate (nuclei and large fragments of the plasma membrane) was further fractionated by isopycnic centrifugation using a sucrose stepwise gradient (46%–42%–29%) for 4 h at 4 °C, 4000× *g* in bucket rotor 4400, Allegra X-30 (Beckman, USA). Then three fractions were collected: nuclei, sediment to the bottom through a 46% sucrose cushion, a fraction located on the border between 46% and 42% sucrose, and a fraction that localized under 29% sucrose. In the collected fractions, the sucrose concentration was adjusted to 0.25 M with buffer A and the organelles were precipitated by centrifugation for 20 min 12,000× *g*. 

### 2.6. Western Blot (WB) Analysis

Cells were lysed in RIPA lysis buffer (ThermoFisher Scientific, Waltham, MA, USA) and samples equable for protein were fractioned by electrophoresis in 8% polyacrylamide gel (PAG) with SDS. The protein transfer, control for the quality of transfer with Ponceau S staining, blocking with 5% non-fat milk in PBST, blotting with primary and secondary antibodies, and visualization of the immune complexes were described previously [18]. Hybond ECL nitrocellulose membrane, ECL reagent, ECL hyperfilm (GE Healthcare, Chicago, IL, USA), and horseradish peroxidase-conjugated goat anti-rabbit secondary antibodies (ab6721, Abcam, Cambridge, UK, or sc-2020, Santa Cruz Biotechnology) were used for the WB analysis. In work rabbit antibodies to SOD1 (ab16831, Abcam), cytochrome-c-oxidase subunit 4 (ab16056, Abcam), NF-κB (p65) (8242, Cell Signaling Technology, Danvers, MA, USA), HIF1α (ab216842, Abcam), and COMMD1 (sc-107497, Santa Cruz Biotechnology) were used as primary antibodies. 

### 2.7. The Content of Ceruloplasmin Protein Secreted by Cells 

The content of ceruloplasmin protein secreted by cells in the medium was valued by immunoblotting (WB). Twenty-five microliters of cultured medium were separated on 8% PAG by non-denaturing electrophoresis according to the Laemmli method. In the study, non-commercial antibodies to high purity human ceruloplasmin were used [19]. 

### 2.8. Preparation the Growth Medium Saturated with Silver Ions

The portions of AgCl crystals were added to RPMI medium with stirring until AgCl crystals ceased to dissolve. Ag-medium was clarified by centrifugation at 10,000× *g*, 1 h. This medium was considered as saturated with Ag(I) growth medium, in which silver atoms are coordinated. Silver concentration in the Ag-saturated medium was determined by atomic absorption spectrometry. It was 150 µM. 

### 2.9. Measurement of Metal Concentration 

In order to reconstruct the copper/silver distribution between the cell compartments, isolated subcellular fractions (see Section 2.5) were resuspended in a volume equal to the volume of cells taken to isolate them. It was considered that cytosol was diluted seven times during cell homogenization. The samples were dissolved in pure concentrated HNO_3_ during 24 h at 56 °C. Metal concentration was measured by graphite furnace atomic absorption spectrometry (FAAS) with electrothermal atomization and Zeeman correction of nonselective absorption on a ZeeNit P650 spectrometer (Analytik Jena, Jena, Germany) with automatic sampling duplication.

### 2.10. Statistical Analysis

The statistical analysis was performed using GraphpadPrism version 6.07 (San Diego, CA, USA). Differences between groups were considered statistically significant when the *p*-values were ≤ 0.05.

## 3. Results

Using H1299 cells, we generated *CTR1* and *DMT1* single KO clones using CRISPR-Cas9 system. Two independent homozygous KO clones for each gene were selected (clone 45 and 46 for *CTR1* and clones 20 and 31 for *DMT1*). From a CTR1 KO clone (clone 45) the second round of transfection, following excision of puromycin and RFP genes by Cre recombinase, to generate CTR1/DMT1 double KO. These last clones were less efficiently generated compared to single KO genes. In fact, while we could isolate several *CTR1*^−/−^ or *DMT1*^−/−^ clones, we were able to isolate one *CTR1*^−/−^, *DMT1*^+/−^ (DKO2) clone and one *CTR1^−/−^*, *DMT1*^−/−^ clone only (DKO1). The experiments were performed in one *CTR1*^−/−^ clone 45, one *DMT1*^−/−^ clone 31 and one DKO (DKO1) clone (Table 2), and some results were confirmed in the second generated clone for each genotype.

Selected clones were initially tested for their ability to grow in vitro. Table 2 reports the doubling times (DT) calculated on three independent experiments, each consisting of six replicates. As it can be seen the selected clones have similar DT all growing slightly faster than parental H1299 cells.

**Figure 1:**
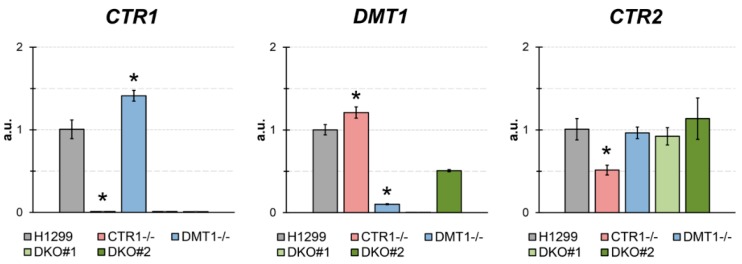
The estimates of expression of CTR1, DMT1, and CTR2 by qRT-PCR in parental H1299 cells, in one clone KO for CTR1 (clone 45), one clone KO for DMT1 (clone 31), clone *CTR1*^−/−^, *DMT1*^+/−^ (DKO2), and clone *CTR1*^−/−^, *DMT1*^−/−^ (DKO1). Y-axis: mRNA fold over actin.

A clear lack of expression of the respective target genes is seen for single KO cells (Figure 1). DKO1 clone showed a dual inhibition of expression, and DKO2 clone, which is *DMT1*^−/−^ heterozygous clone, DMT1 expression was higher than in DMT1 KO or DKO1. Interestingly, CTR1 KO clone showed a consistent increase in DMT1 expression (Figure 1), while in the DMT1 KO clone, the expression of CTR1 was slightly higher than in the parental cells. In cells that do not produce CTR1, the expression of the *CTR2* gene, compared with the parent strain, was strongly decreased and did not change in DMT1-deficient clones (Figure 1).

**Figure 2:**
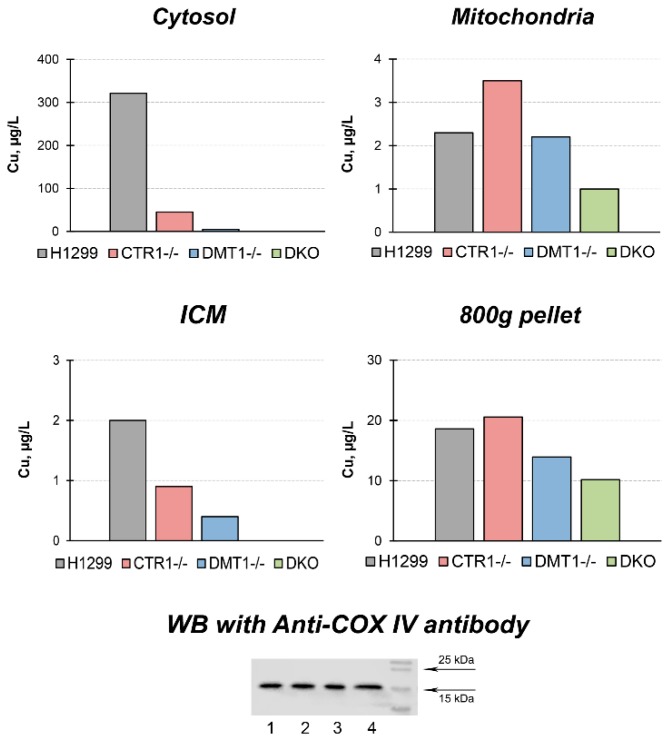
Copper distribution in CTR1 KO, DMT1 KO, and DKO cells. Y-axis: copper concentration (µg/L) in subcellular fractions. Down below: WB with antibodies to Cox4. Lines: 1, H1299 2, CTR1 KO; 3, DMT1 KO; 4, DKO. Each sample contained 30 µg protein.

The effects of the single and double knockout of *CTR1* and *DMT1* on copper distribution in the cells are shown in Figure 2. In cytosol of CTR1 KO, the copper level decreased by a factor of six. In DMT KO clone, the copper content was even lower, and in DKO, the content of copper dropped further below the threshold of reliable measurements. In the ICM, the copper level in CTR1 KO decreased almost twofold, in DMT KO, it was roughly 1/8 of that of parental cells, while in the DKO clone the levels of copper in ICM were below the detection level. In the mitochondria of cells with single knockouts, the copper content remained almost unchanged, but it sharply decreased in DKO cells. At the same time, the level of the cytochrome-c-oxidase subunit 4 (Cox4) remained unchanged in the cells of the all derived clones (Figure 2). The copper concentration in a low-speed pellet (nuclei, 800× *g*) was lower in DMT1 and DKO cells, but this decline was not dramatic (Figure 2).

**Figure 3:**
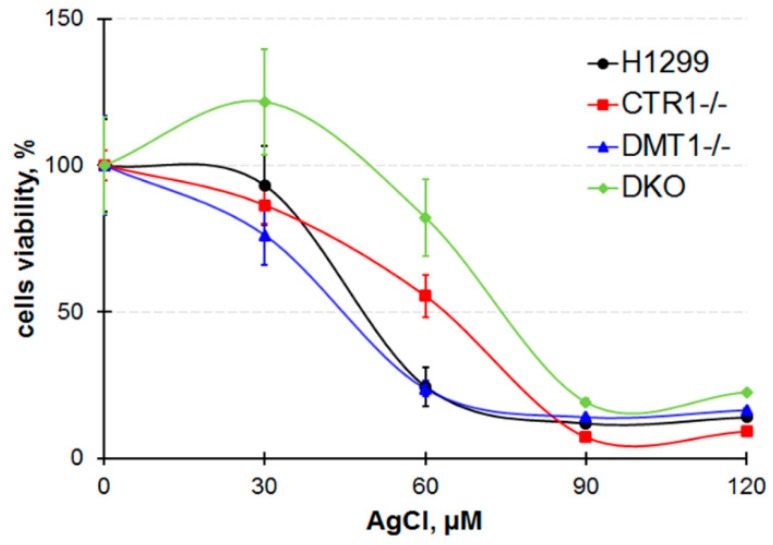
Cells viability after treatment in medium with AgCl for 72 h.

**Figure 4:**
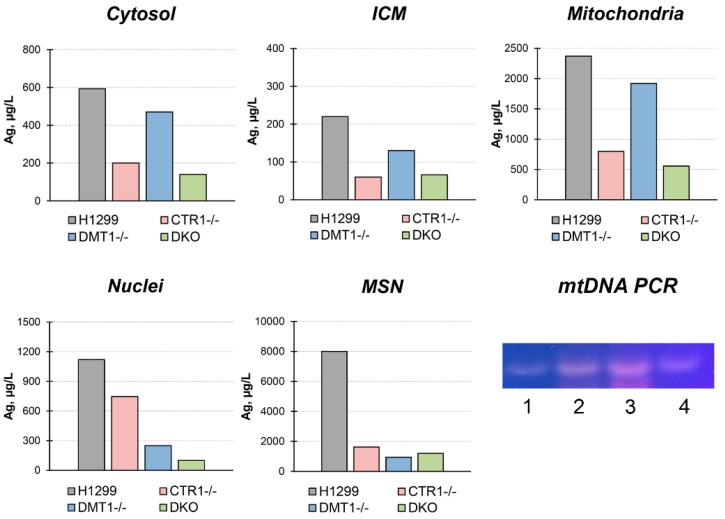
Silver distribution in H1299, CTR1 KO, DMT1 KO, and DKO cells. Y-axis: copper concentration (µg/L) in subcellular fractions. Gel: mtDNA-specific PCR products in subcellular fractions isolated from H1299 cells. 1, nuclei; 2, MSN; 3, mitochondria; 4, ICM. The samples contained 10 µ protein.

To trace the distribution of Cu(I) we use silver ions (Ag(I)) because they are isoelectronic to Cu(I) thence bind with Cu(I)-transporters. Moreover, there is no physiologically role of silver in the cell and it is easier to follow its intracellular routes. At first, the cells were tested for the toxic effect of silver ions. The data in Figure 3 demonstrate that 30 µM silver ions weakly influenced the viability of cells while 90 µM of silver ions were highly toxic to all cells. However, at 60 µM Ag(I), cells not expressing CTR1 gene were more resistant to silver ions than wild-type (WT) cells or DMT1 KO clones. In these clones, the distribution of silver in subcellular compartments was traced at 30 µM of Ag(I) concentration in the medium. The data are presented in Figure 4. They show that silver is predominantly accumulated in the mitochondria. In CTR1 KO and DKO clone compartments, silver concentration was three to five times lower.

In DMT1 KO clones, silver content was higher than in CTR1 KO and DKO clones, but lower than WT cells. In the pellet forming at low sedimentation speed (800× *g*, 10 min), we found silver in extremely high concentration. Because this pellet includes nuclei and large fragments of the plasma membrane, we fractioned it in the sucrose gradient (see “Methods”). In the fraction that does not sediment through a 28% sucrose solution (ρ = 1.1175 g/L), so it corresponds in density to the plasma membrane, atomic silver is practically absent (data not shown). In the nuclei, the fraction that sedimented through 46% sucrose (density more 1.2079 g/L), the silver concentration was twofold lower than in mitochondria. In addition, the visually detectable fraction at the border of 46% sucrose was collected. According to its density, it was designated as mitochondria co-sedimented with nuclei (MSN). The bulk of the silver was accumulated in this fraction (Figure 4).

MSN silver concentration was higher than in other subfractions, and it was higher in parent H1299 cells than in derived clones. In all subcellular fractions isolated from WT H1299, the presence of mtDNA was verified by PCR analysis. The data presented in Figure 4 (gel) show that the bulk of mtDNA is found in the mitochondrial fraction, but it is also present in MSN and ICM and as a trace in nuclei.

**Figure 5:**
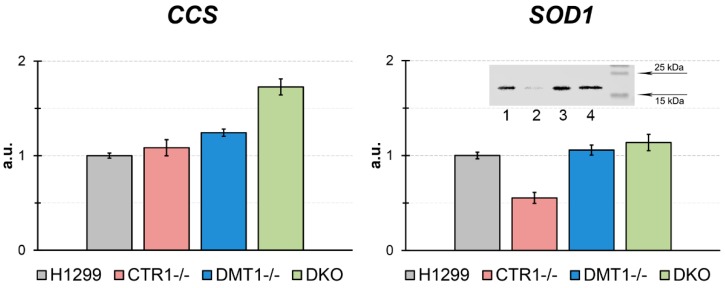
CCS and SOD1 gene expression in the KO cells derived from H1299. Y-axis: mRNA levels (fold over actin). * *p* < 0.05 compared with H1299. Y-axis: mRNA fold change. Insert: WB for SOD1. 1, H1299; 2, CTR1 KO; 3, DMT1 KO; 4, DKO. Each sample contained 30 µg protein.

In derived clones, the expression of *CCS* (copper chaperone for SOD1) and *SOD1* (Cu,Zn-superoxide dismutase, intracellular cuproenzyme) genes were tested (Figure 5). The *CCS* gene expression level in DMT1 KO and DKO cells significantly increased (Figure 5). In CTR1 KO, the concentration of SOD1 mature transcripts was lower as compared to parent H1299, however in DMT1 KO and DKO clones, SOD1-mRNA level coincided with those in the H1299 cells (Figure 5). The SOD1 protein level decreased in CTR1 KO cells and did not change in DMT1-deficient clones. It was corresponding to SOD1-mRNA concentration (Figure 5).

**Figure 6:**
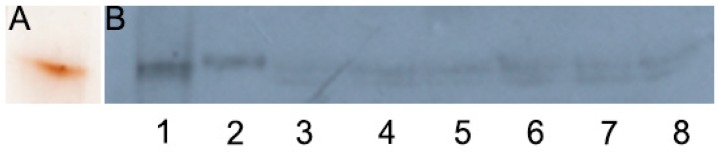
Ceruloplasmin gene activity in KO cells derived from H1299. (**A**) Human serum, 0.025 µL per line, gel was stained with *orto*-dianisidine, (**B**) WB with antibodies to human ceruloplasmin: 1, human serum, 0.01 µL per line; 2, H1299; 3, CTR1 KO45; 4, CTR1 KO46; 5, DMT1 KO31; 6, DMT1 KO20; 7, DKO1; 8, DKO2; 25 µL medium per line. The proteins were separated in 8% PAG without SDS by electrophoresis.

Using ceruloplasmin as an example, we checked the ability of the derived clones to synthesize secretory cuproenzymes. Ceruloplasmin, a polyfunctional blue multicopper (ferr)oxidase of hepatic origin, is the main copper-containing blood serum protein [20,21,22,23]. It has been shown that ceruloplasmin is also synthesized in the lungs [24]. The physiological role of lung-specific ceruloplasmin has not been established. The data in Figure 6 shows that WT H1299 cells synthesize and secrete ceruloplasmin, which corresponds to serum holo-ceruloplasmin by its mobility. In all derived cells, the synthesis of immunoreactive ceruloplasmin decreased.

**Figure 7:**
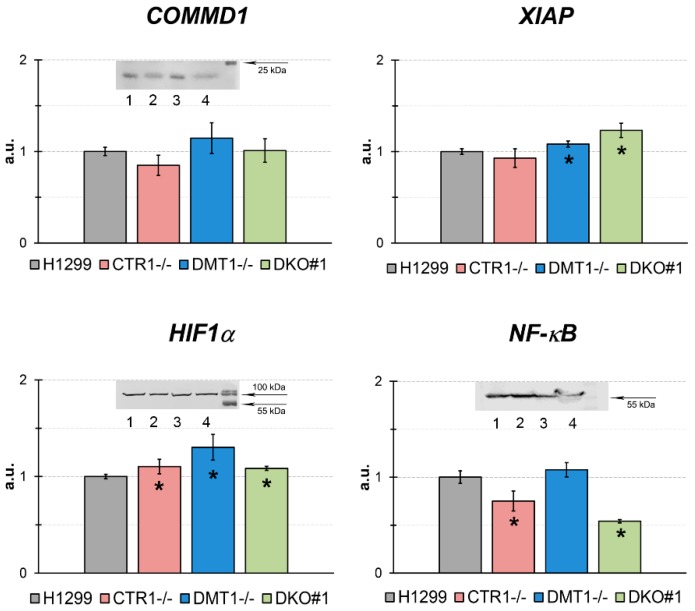
qRT-PCR analysis of concentrations of mature mRNA, which are the products of genes related to the copper regulatory role in the different H1299-derived clones. Y-axis: mRNA levels, a.u. (fold over actin). * *p* < 0.05 compared with H1299. (**Inserts**) WB analysis: COMMD1, each sample contained 80 µg protein. HIF1α, each sample contained 50 µg protein, and NF-*κ*B (p65), each sample contained 50 µg protein. In WB: 1, H1299; 2, CTR1 KO; 3, DMT1 KO; 4, DKO.

We next evaluated the pattern of gene expression for selected genes known to be linked to copper regulation and, more generally, to copper homeostasis (Figure 7).

We tested the expression of *COMMD1* (copper metabolism domain containing 1), *XIAP* (X-linked inhibitor of apoptosis protein), *HIF1α* (hypoxia-inducible factor 1-alpha), and *NF-κB* (nuclear factor kappa-light-chain-enhancer of activated B cells) genes. The expression of *COMMD1* gene was significantly increased in DMT1 KO cells only. These data agreed with WB analysis for COMMD1. The HIF1α-mRNA and protein concentrations were not significantly changed. XIAP gene expression was increased only in DKO cells. The mRNA level of NF-κB p65 subunit was decreased in DKO cells and that was supported by the WB data.

**Figure 8:**
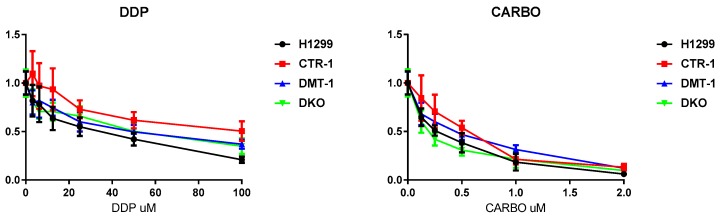
Cytotoxic activity of cisplatin and carboplatin in H1299 parental cells and in KO derived. X-axis: drug concentration. Y-axis: growth rate. Growth inhibitory activity was determined using the MTS test. Each point represents the mean ± SD of six replicates.

**Figure 9:**
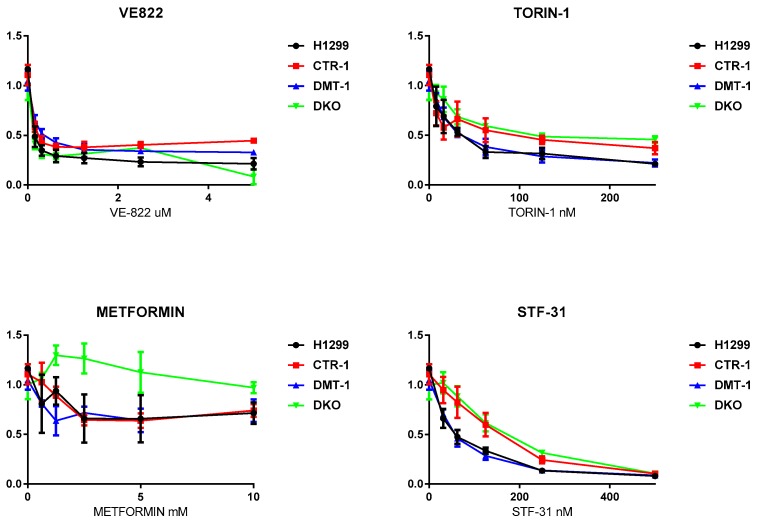
Cytotoxic activity of VE-822, Torin-1, Metformin, and STF-3 in H1299 parental cells and in KO derived. X-axis: drug concentration. Y-axis: growth rate. Growth inhibitory activity was determined using the MTS test. Each point represents the mean ± SD of six replicates.

Finally, the clones were tested for their ability to respond to cytotoxic compounds, including cisplatin, carboplatin, VE-822, Torin-1, metformin, and STF-31. The obtained results are presented in Figure 8 and Figure 9. CTR1 KO cells were significantly more resistant to cisplatin (known substrate of CTR1 [11]) than parental H1299 cells in the range of high cisplatin concentrations (Figure 8). The other clones displayed cisplatin sensitivity similar to parental cells. CTR1 KO cells did not demonstrate resistance to carboplatin (cisplatin analog).

VE-822, an inhibitor of the stress response ATR signaling pathway in DNA repair [25], TORIN-1, mTOR inhibitor, a member of phosphatidylinositol 3-kinase-related kinases family, which functions as a serine/threonine protein kinase in a different signaling way [26], displayed similar toxicity for all the clones (Figure 9). The toxic effect of STF-31, specific glucose transporter 1 inhibitor [27], was reduced in CTR1-/- and DKO cells (Figure 9). Meanwhile, interesting results were obtained with the AMPK inhibitor metformin, which decreases blood glucose concentration and is widely used in the treatment of obesity and in some types of cancer [28]. With this drug parental cells and the single KO (both CTR1 and DMT1) behaved similarly. The DKO clone was instead notably less responsive.

## 4. Discussion

The aim of this study was to generate and characterize the *CTR1* and *DMT1* single knockout and double *CTR1*/*DMT1* knockout clones and to evaluate them as a potential model for the studies of copper metabolism in catalytic and regulatory copper pools. Moreover, CTR1 is an important member in the system of maintaining the balance of copper and cisplatin uptake [12]. High CTR1 level predicts prolonged survival and enhanced response to chemotherapy in cancer patients [29]. The ability to manipulate the activity of this gene can help to develop approaches to reduce the growth rate of tumors through controlling neovascularization and/or increasing the effectiveness of cisplatin use. Thus, the information obtained on *CTR1* gene knockout in different cell lines is useful to optimized chemotherapy in cancer. We showed that the doubling time of single KO clones derived from H1299 was similar to that of parent cells. Therefore, these cells are convenient for laboratory studies. There are contradictory data on whether the knockout of the CTR1 gene affects the cell growth rate. In human umbilical vein endothelial cells, CTR1 KO decreased proliferation almost twofold [30]. At the same time in HEK293T and OVCAR8 lines, CTR1 KO had a slightly decreased rate of growth and most have similar growth rates to the parental cells once they reach a threshold cell density [31]. Perhaps, the conflicting in vitro data may arise from cell-specific copper metabolism.

The traces of CTR1-mRNA, or DMT1-mRNA, were detected in CTR1 KO clones, or DMT1 KO clones correspondingly (Figure 1). Meanwhile, the concentration of DMT1-mRNA increased by a factor of about 1.5 in CTR1 KO clones. In DMT1 KO clone the concentration of CTR1-mRNA weakly increased (but *p* < 0.05). These results generally agree with the observation that DMT1 can compensate for the loss of CTR1 function and restore copper balance in the cell and *vice versa* [17]. In DKO clones, both CTR1 and DMT1 mRNA levels were almost absent, except for DMT1 in DKO heterozygous clone, in which some DMT1 expression was detectable. What is more, we checked whether *CTR1* or *DMT1* gene loss affects the expression of *CTR2* gene, encoding low-affinity copper transporter, which is structurally similar and highly related to CTR1 protein [32,33,34]. Unlike CTR1, CTR2 lost the ability to transport copper ions from the environment, it regulates the flow of copper through the cleavage of the CTR1 ectodomain and participates in the copper mobilization from organelles to the cytosol [35]. What is more, CTR2 takes part in limiting cisplatin accumulation [36]. CTR1 KO clone does not affect the activity of the *CTR2* gene, but in cells which do not express *DMT1* the *CTR2* gene activity increases significantly (Figure 1). In the absence of additional research, we can assume that CTR2 is involved in the distribution of copper, which is imported through CTR1-independent way. Possibly, CTR2 can accept coordinated copper, but not copper ions. It cannot be absolutely excluded that CTR2 imports copper. This cautious assumption is based on the fact that all methionine residues necessary for the import of copper and the functional cuprophilic pore are present in CTR2 [33].

The loss of function of *CTR1* or *DMT1* genes in single KO clones led to a copper deficiency in the cells and changed intracellular copper distribution (Figure 2). The copper levels in cytosol were strongly decreased as compared to parent line, but they were higher in CTR1 KO cells than in DMT1 KO cells, again in line with the ability of DMT1 gene to compensate CTR1 function [17]. It is possible that the decrease of copper levels in cytosol corresponds to the depletion of Cu-metallothionein [37]. In mitochondria, copper concentrations did not dramatically change as compared to the parent line. Perhaps in mitochondria, copper is transported by two proteins: from cytosol to mitochondrial intermembrane space by DMT1 localized in outer membrane [38] and from intermembrane space to matrix by the phosphate carrier SLC25A3 [39]. Because mitochondria of DMT1 KO cells did not lose copper completely, we think that there is yet unidentified carrier of copper in the mitochondrial outer membrane. This assumption was made within the framework of modern concepts that the mitochondrial copper requirements are of highest priority for the cell [40]. Moreover, it is supported by our data showing that the nuclear subunit Cox4 level, whose expression is controlled by copper-dependent catalytic core assembly of the complex IV [41], did not change in the derived clones (Figure 2). This allows us to think that putative copper bound to methanobactin-like substance is an important participant of the mitochondrial/cytosolic copper homeostatic system [42,43].

In ICM, copper concentration decreased by a factor of 2 and 4 in CTR1 KO and DMT1 KO clones, respectively (Figure 2). In accord with the decrease of copper concentration in the ICM, where the ceruloplasmin is metallated [44], the ceruloplasmin protein concentration in the medium decreased too (Figure 6). Interestingly, that strong restriction of copper intake by Golgi complex leads to the same phenotype as in patients with Wilson disease, which develops from mutations in the *ATP7B* gene, but not in ceruloplasmin gene [45].

In all derived clones, the nuclear copper concentration almost corresponded to that in the parent line (Figure 2). Our results are consistent with the data obtained by Lin et al., which showed that CTR1/DMT1 KO cells retain the intracellular copper level close to the parent strains [17]. Moreover, Bompiani et al. demonstrated that CTR1 KO cells derived from HEK-293T and OVCAR8 cells displayed small changed in basal Cu levels [31]. These results further support the idea that cells have an unidentified pathway for copper uptake. The role of nuclei as dynamic copper storage in vivo was demonstrated in newborn rats [18] and Atp7b^−/−^ mice [46].

We then used silver ions to compare the metal-transporting properties of CTR1 and DMT1 and to evaluate the difference between Cu(I) and Cu(II) transport routes. Cu(I) and Ag(I) have similar ion radii and the same structure of valence shells, so abiogenic Ag(I) is captured by Cu(I)-binding sites in CTR1 N-terminal domain and effectively transported into the cell. Accumulation of silver ions eventually leads to cell death [47]. Unlike CTR1, DMT1 does not contain thioether-rich metal-binding sites, so we supposed that CTR1-negative clones (CTR1 KO and homozygous DKO) would display increased resistance to silver ions as compared to wild type cells. Indeed, the cells that did not express CTR1 were more resistant to the toxic effects of silver ions (Figure 3). Still DKO clone was the most resistant, indirectly proving that DMT1 can transfer both, Cu(II) and Cu(I)/Ag(I) [16]. This conclusion is supported by data that cells that have lost *CTR1* or *DMT1* genes accumulated silver (Figure 4). The distribution of silver in the cells of the parent line (Figure 4) was in good agreement with known effects of Ag(I) binding by metallothionein [48], its accumulation in mitochondria [49] and its translocation to the lumen of Golgi complex [50]. Unexpectedly, in the low-rate sedimentary fraction, extremely high silver concentration was found. About 10% of this silver pool was found in the nuclear fraction, the remaining silver was accumulated in the fraction with a density close to mitochondria. Preliminarily, by the presence of mtDNA, this fraction was suspected by us to be mitophagosomes (we understand that the assumption still needs special investigation). The association of silver with MSN (“mitophagosomes”) is consistent with the idea that mitochondria are involved in the detoxification of copper excess through induction of autophagy [51]. We also found mtDNA in ICM fraction (Figure 4). These results agree with the data that mitochondria tightly contact with the endoplasmic membrane in mitophagosome processing, calcium homeostasis, and lipid trafficking [52,53].

In obtained clones, expression of SOD1, the key enzyme of the antioxidant cellular system, and CCS, an essential copper chaperone for SOD1 [54], were tested (Figure 5). The SOD1-mRNA concentration and SOD1 protein level were decreased in CTR1 KO clone only. Meanwhile, in this clone, the activity of CCS gene did not change. Now we cannot explain, why CTR1 deficiency did not activate CCS gene expression. On the contrary, in DMT1-deficiency cells, where cytosolic copper concentration dramatically dropped, the CCS gene expression increased, and SOD1 expression on the transcription and translation levels were supported at the same those in the parental cells. These results agree with data that low copper status results in decreased SOD1 level and increased *CCS* gene expression [55].

Moreover, the expression levels of genes, whose activity is linked or supposed to be linked to copper regulatory function, were analyzed in the cells with the loss of CTR1 and/or DMT1 function (Figure 7). We did not find striking changes in expression of *COMMD1*, *HIF1α*, *NF-κB*, and *XIAP* genes in KO and DKO lines. However, the selective effect of CTR1 and DMT1 loss on the expression of these genes can be noticed. So, the loss of *CTR1* resulted in decreased levels of COMMD1, XIAP, and NF-κB. Differently, the *DMT1* deficiency induced the increase of COMMD1, HIF1α, and XIAP levels.

Tests for the sensitivity of the obtained cells to various drugs proved the fact, that the loss of *CTR1*, but not *DMT1*, rendered the cells resistant to cisplatin (Figure 8) [56]. Other drugs, which were tested in the present work (Figure 9), affected the viability of the CTR1 KO, DMT1 KO, DKO and WT cells in a similar manner. The only clear exception was metformin: DKO cells displayed resistance when exposed to this drug. Metformin has a very diverse spectrum of interactions [28], including the ability to bind and transfer copper atoms [57], the latter may enhance the viability of copper-deficient DKO cells. As metformin is being widely used for different purposes, including cancer treatment, the knowledge that copper levels or transport can modify its activity could have if confirmed important implications.

## 5. Conclusions

The main lessons of our work are the following. In addition to CTR1 and DMT1, there are alternative way(s) to transfer copper into the cell. Possible, it is an ion exchanger [58]. Besides DMT1, the mitochondrial outer membrane contains an unidentified importer of copper. The basal level of intracellular copper in cells, which have lost CTR1 or DMT1 changes slightly when compared to the parent strain [17,31] and our data, but the intracellular copper distribution changes significantly: cytosol and ICM almost completely lose copper, but the mitochondria from single KO keep copper at levels close to WT H1299. With a strict restriction of the copper influx, the nuclear copper pool changes slightly. DMT1-mediated copper transport provides the formation of vital intracellular cuproenzymes (SOD1, COX) and secretory cuproenzymes. Therefore, cuproenzyme-specific cytosolic chaperones can receive copper not only from the C-domain of CTR1, but also from DMT1, possibly through intermediaries that have not yet been identified or from metallothionein redox cycle [59]. It appears that the vital cuproenzymes metalation is more important for the cell than the formation of the secretory Cu-required holo-enzymes. The difference in the distribution of copper/silver in subfractions of cells that have lost CTR1 or DMT1 is not clearly manifested, but the role of mitochondria as important organizers of copper metabolism [60] as well as nuclei is obvious. We have not received obvious evidence that the pool of regulatory copper enters the cells separately from the catalytic pool. However, the selective response of COMMD1, XIAP, HIF1α and NF-κB genes to CTR1- or DMT1-dependent copper deficiency allow us to suggest that isogenic cell lines with differential knockouts of copper transporters and discriminated copper import pathways will be useful for studies of the regulatory role of copper and could also have important translational relevance.

## Figures and Tables

**Table 1:** Primer sequences used for qRT-PCR analysis.

Gene	Primers	Direct
CTR1	5′-GGAGGAGACAGCAGCATGAT-3′ 3′-GTCGACCTCTTTACCGACCT-5′	Forward Reverse
DMT1	5′-GTCCTCATGCTGGCCTCTTT-3′ 3′-AGATGTAAACCCGTCACCCC-5′	Forward Reverse
CTR2	5′-GCGGTGCTTCTGTTTGATTTCT-3′ 3′-ACTTCCGTAGTTCCAACCGT-5′	Forward Reverse
SOD1	5′-CCAGTGCAGGTCCTCACTTT-3′ 3′-CGACTGTTTCTACCACACCG-5′	Forward Reverse
CCS	5′-CGGTACTCAAGGGCATGGG-3′ 3′-AAGGATGTCGACTGGGGACT-5′	Forward Reverse
HIF1α	5′-GCTTTAACTTTGCTGGCCCC-3′ 3′-ACTACTGGTCGTTGAACTCCT-5′	Forward Reverse
XIAP	5′-ACTCAGTTAACAAGGAGCAGCT-3′ 3′-AAGTGACACCTCCTCCCGAT-5′	Forward Reverse
NF-κB	5′-CTGCTGGACCCAAGGACATG-3′ 3′-GTCTCCGCACATATTCCCCG-5′	Forward Reverse
COMMD1	5′-GGCGCTGTGGGTGAAGAG-3′ 3′-CTGTACCTAAAGTTGGTCGACC-5′	Forward Reverse

**Table 2:** Doubling time of H1299-derive clones.

Cell Line	Doubling Time, (h) ± SD
H1299	18.77 ± 1.49
CTR1 KO45	15.03 ± 4.09
DMT1 KO31	13.45 ± 2.31
DKO2 (CTR1^−/−^, DMT1^+/−^)	14.51 ± 3.30
DKO1 (CTR1^−/−^, DMT1^−/−^)	15.36 ± 2.56

## References

[1] Vonk W.I., Wijmenga C., van de Sluis B. (2008). Relevance of animal models for understanding mammalian copper homeostasis. Am. J. Clin. Nutr..

[2] Ridge P.G., Zhang Y., Gladyshev V.N. (2008). Comparative genomic analyses of copper transporters and cuproproteomes reveal evolutionary dynamics of copper utilization and its link to oxygen. PLoS ONE.

[3] Li S., Zhang J., Yang H., Wu C., Dang X., Liu Y. (2015). Copper depletion inhibits CoCl2-induced aggressive phenotype of MCF-7 cells via downregulation of HIF-1 and inhibition of Snail/Twist-mediated epithelial-mesenchymal transition. Sci. Rep..

[4] Hou M.M., Polykretis P., Luchinat E., Wang X., Chen S.N., Zuo H.H., Yang Y., Chen J.-L., Ye Y., Li C. (2017). Solution structure and interaction with copper in vitro and in living cells of the first BIR domain of XIAP. Sci. Rep..

[5] Riera-Romo M. (2018). COMMD1: A multifunctional regulatory protein. J. Cell. Biochem..

[6] Bartuzi P., Hofker M.H., van de Sluis B. (2013). Tuning NF-κB activity: A touch of COMMD proteins. Biochim. Biophys. Acta.

[7] Ostrakhovitch E.A., Song Y.P., Cherian M.G. (2016). Basal and copper-induced expression of metallothionein isoform 1,2 and 3 genes in epithelial cancer cells: The role of tumor suppressor p53. J. Trace Elem. Med. Biol..

[8] Bhattacharjee A., Chakraborty K., Shukla A. (2017). Cellular copper homeostasis: Current concepts on its interplay with glutathione homeostasis and its implication in physiology and human diseases. Metallomics.

[9] Kardos J., Héja L., Simon Á., Jablonkai I., Kovács R., Jemnitz K. (2018). Copper signalling: Causes and consequences. Cell Commun. Signal..

[10] Sharp P.A. (2003). Ctr1 and its role in body copper homeostasis. Int. J. Biochem. Cell Biol..

[11] Kilari D., Guancial E., Kim E.S. (2016). Role of copper transporters in platinum resistance. World J. Clin. Oncol..

[12] Lasorsa A., Natile G., Rosato A., Tadini-Buoninsegni F., Arnesano F. (2018). Monitoring interactions inside cells by advanced spectroscopies: Overview of copper transporters and csplatin. Curr. Med. Chem..

[13] Gunshin H., Mackenzie B., Berger U.V., Gunshin Y., Romero M.F., Boron W.F., Nussberger S., Gollan J.L., Hediger M.A. (1997). Cloning and characterization of a mammalian proton-coupled metal-ion transporter. Nature.

[14] Czachorowski M., Lam-Yuk-Tseung S., Cellier M., Gros P. (2009). Transmembrane topology of the mammalian Slc11a2 iron transporter. Biochemistry.

[15] Wang D., Song Y., Li J., Wang C., Li F. (2011). Structure and metal ion binding of the first transmembrane domain of DMT1. Biochim. Biophys. Acta.

[16] Arredondo M., Munoz P., Mura C.V., Nunez M.T. (2003). DMT1, a physiologically relevant apical Cu1+ transporter of intestinal cells. Am. J. Physiol. Cell Physiol..

[17] Lin C., Zhang Z., Wang T., Chen C., Kang Y.J. (2015). Copper uptake by DMT1: A compensatory mechanism for CTR1 deficiency in human umbilical vein endothelial cells. Metallomics.

[18] Zatulovskaia Y.A., Ilyechova E.Y., Puchkova L.V. (2015). The features of copper metabolism in the rat liver during development. PLoS ONE.

[19] Sokolov A.V., Kostevich V.A., Romanico D.N., Zakharova E.T., Vasilyev V.B. (2012). Two-stage method for purification of ceruloplasmin based on its interaction with neomycin. Biochemistry.

[20] Bielli P., Calabrese L. (2002). Structure to function relationships in ceruloplasmin: A ‘moonlighting’ protein. Cell. Mol. Life Sci..

[21] Golenkina E.A., Viryasova G.M., Galkina S.I., Gaponova T.V., Sud’ina G.F., Sokolov A.V. (2018). Fine regulation of neutrophil oxidative status and apoptosis by ceruloplasmin and its derivatives. Cells.

[22] Samygina V.R., Sokolov A.V., Bourenkov G., Schneider T.R., Anashkin V.A., Kozlov S.O., Kolmakov N.N., Vasilyev V.B. (2017). Rat ceruloplasmin: A new labile copper binding site and zinc/copper mosaic. Metallomics.

[23] Bernevic B., El-Khatib A.H., Jakubowski N., Weller M.G. (2018). Online immunocapture ICP-MS for the determination of the metalloprotein ceruloplasmin in human serum. BMC Res. Notes.

[24] Fleming R.E., Whitman I.P., Gitlin J.D. (1991). Induction of ceruloplasmin gene expression in rat lung during inflammation and hyperoxia. Am. J. Physiol..

[25] Rahal O.N., Fatfat M., Hankache C., Osman B., Khalife H., Machaca K., Muhtasib H.G. (2016). Chk1 and DNA-PK mediate TPEN-induced DNA damage in a ROS dependent manner in human colon cancer cells. Cancer Biol. Ther..

[26] Hare S.H., Harvey A.J. (2017). mTOR function and therapeutic targeting in breast cancer. Am. J. Cancer Res..

[27] Chan D.A., Sutphin P.D., Nguyen P., Turcotte S., Lai E.W., Banh A., Reynolds G.E., Chi J.T., Wu J., Solow-Cordero D.E. (2011). Targeting GLUT1 and the Warburg effect in renal cell carcinoma by chemical synthetic lethality. Sci. Transl. Med..

[28] Schulten H.J. (2018). Pleiotropic effects of metformin on cancer. Int. J. Mol. Sci..

[29] Sun S., Cai J., Yang Q., Zhao S., Wang Z. (2017). The association between copper transporters and the prognosis of cancer patients undergoing chemotherapy: A meta-analysis of literatures and datasets. Oncotarget.

[30] Narayanan G., Bharathidevi S.R., Vuyyuru H., Muthuvel B., Konerirajapuram Natrajan S. (2013). CTR1 silencing inhibits angiogenesis by limiting copper entry into endothelial cells. PLoS ONE.

[31] Bompiani K.M., Tsai C.Y., Achatz F.P., Liebig J.K., Howell S.B. (2016). Copper transporters and chaperones CTR1, CTR2, ATOX1, and CCS as determinants of cisplatin sensitivity. Metallomics.

[32] Wee N.K., Weinstein D.C., Fraser S.T., Assinder S.J. (2013). The mammalian copper transporters CTR1 and CTR2 and their roles in development and disease. Int. J. Biochem. Cell Biol..

[33] Öhrvik H., Thiele D.J. (2015). The role of Ctr1 and Ctr2 in mammalian copper homeostasis and platinum-based chemotherapy. J. Trace Elem. Med. Biol..

[34] Logeman B.L., Wood L.K., Lee J., Thiele D.J. (2017). Gene duplication and neo-functionalization in the evolutionary and functional divergence of the metazoan copper transporters Ctr1 and Ctr2. J. Biol. Chem..

[35] van den Berghe P.V., Folmer D.E., Malingré H.E., van Beurden E., Klomp A.E., van de Sluis B., Merkx M., Berger R., Klomp L.W.J. (2007). Human copper transporter 2 is localized in late endosomes and lysosomes and facilitates cellular copper uptake. Biochem. J..

[36] Blair B.G., Larson C.A., Safaei R., Howell S.B. (2009). Copper transporter 2 regulates the cellular accumulation and cytotoxicity of cisplatin and carboplatin. Clin. Cancer Res..

[37] Calvo J., Jung H., Meloni G. (2017). Copper metallothioneins. IUBMB Life.

[38] Wolff N.A., Ghio A.J., Garrick L.M., Garrick M.D., Zhao L., Fenton R.A., Thévenod F. (2014). Evidence for mitochondrial localization of divalent metal transporter 1 (DMT1). FASEB J..

[39] Boulet A., Vest K.E., Maynard M.K., Gammon M.G., Russell A.C., Mathews A.T., Cole S.E., Zhu X., Phillips C.B., Kwong J.Q. (2018). The mammalian phosphate carrier SLC25A3 is a mitochondrial copper transporter required for cytochrome *c* oxidase biogenesis. J. Biol. Chem..

[40] Dodani S.C., Leary S.C., Cobine P.A., Winge D.R., Chang C.J. (2011). A targetable fluorescent sensor reveals that copper-deficient SCO1 and SCO2 patient cells prioritize mitochondrial copper homeostasis. J. Am. Chem. Soc..

[41] Timón-Gómez A., Nývltová E., Abriata L.A., Vila A.J., Hosler J., Barrientos A. (2018). Mitochondrial cytochrome *c* oxidase biogenesis: Recent developments. Semin. Cell Dev. Biol..

[42] Cobine P.A., Ojeda L.D., Rigby K.M., Winge D.R. (2004). Yeast contain a non-proteinaceous pool of copper in the mitochondrial matrix. J. Biol. Chem..

[43] Cobine P.A., Pierrel F., Bestwick M.L., Winge D.R. (2006). Mitochondrial matrix copper complex used in metallation of cytochrome oxidase and superoxide dismutase. J. Biol. Chem..

[44] Polishchuk R., Lutsenko S. (2013). Golgi in copper homeostasis: A view from the membrane trafficking field. Histochem. Cell Biol..

[45] Schilsky M.L. (2017). Wilson disease: Diagnosis, treatment, and follow-up. Clin. Liver Dis..

[46] Huster D., Finegold M.J., Morgan C.T., Burkhead J.L., Nixon R., Vanderwerf S.M. (2006). Consequences of copper accumulation in the livers of the Atp7b-/- (Wilson disease gene) knockout mice. Am. J. Pathol..

[47] Bertinato J., Cheung L., Hoque R., Plouffe L.J. (2010). Ctr1 transports silver into mammalian cells. J. Trace Elem. Med. Biol..

[48] Palacios O., Polec-Pawlak K., Lobinski R., Capdevila M., González-Duarte P. (2003). Is Ag(I) an adequate probe for Cu(I) in structural copper-metallothionein studies? The binding features of Ag(I) to mammalian metallothionein 1. J. Biol. Inorg. Chem..

[49] Zatulovskiy E.A., Skvortsov A.N., Rusconi P., Ilyechova E.Y., Babich P.S., Tsymbalenko N.V., Broggini M., Puchkova L.V. (2012). Serum depletion of holo-ceruloplasmin induced by silver ions in vivo reduces uptake of cisplatin. J. Inorg. Biochem..

[50] Ilyechova E.Y., Saveliev A.N., Skvortsov A.N., Babich P.S., Zatulovskaia Y.A., Pliss M.G., Korzhevskii D.E., Tsymbalenko N.V., Puchkova L.V. (2014). The effects of silver ions on copper metabolism in rats. Metallomics.

[51] Polishchuk E.V., Polishchuk R.S. (2016). The emerging role of lysosomes in copper homeostasis. Metallomics.

[52] Böckler S., Westermann B. (2014). ER-mitochondria contacts as sites of mitophagosome formation. Autophagy.

[53] Szymanski J., Janikiewicz J., Michalska B., Patalas-Krawczyk P., Perrone M., Ziółkowski W., Duszyński J., Pinton P., Dobrzyń A., Więckowski M.R. (2017). Interaction of mitochondria with the endoplasmic reticulum and plasma membrane in calcium homeostasis, lipid trafficking and mitochondrial structure. Int. J. Mol. Sci..

[54] Wong P.C., Waggoner D., Subramaniam J.R., Tessarollo L., Bartnikas T.B., Culotta V.C., Price D.L., Rothstein J., Gitlin J.D. (2000). Copper chaperone for superoxide dismutase is essential to activate mammalian Cu/Zn superoxide dismutase. Proc. Natl. Acad. Sci. USA.

[55] Spinazzi M., Sghirlanzoni A., Salviati L., Angelini C. (2014). Impaired copper and iron metabolism in blood cells and muscles of patients affected by copper deficiency myeloneuropathy. Neuropathol. Appl. Neurobiol..

[56] Song I.S., Savaraj N., Siddik Z.H., Liu P., Wei Y., Wu C.J., Kuo M.T. (2004). Role of human copper transporter Ctr1 in the transport of platinum-based antitumor agents in cisplatin-sensitive and cisplatin-resistant cells. Mol. Cancer Ther..

[57] Logie L., Harthill J., Patel K., Bacon S., Hamilton D.L., Macrae K., McDougall G., Wang H.H., Xue L., Jiang H. (2012). Cellular responses to the metal-binding properties of metformin. Diabetes.

[58] Zimnicka A.M., Ivy K., Kaplan J.H. (2011). Acquisition of dietary copper: A role for anion transporters in intestinal apical copper uptake. Am. J. Physiol. Cell Physiol..

[59] Kang Y.J. (2006). Metallothionein redox cycle and function. Exp. Biol. Med..

[60] Baker Z.N., Cobine P.A., Leary S.C. (2017). The mitochondrion: A central architect of copper homeostasis. Metallomics.

